# Cooperation on dynamic networks within an uncertain reputation environment

**DOI:** 10.1038/s41598-018-27544-5

**Published:** 2018-06-14

**Authors:** Pablo Lozano, Alberto Antonioni, Marco Tomassini, Angel Sánchez

**Affiliations:** 1 Grupo Interdisciplinar de Sistemas Complejos (GISC), Departamento de Matemáticas, Universidad Carlos III de Madrid, E-28911 Leganés, Madrid Spain; 2 Unidad Mixta de Comportamiento y Complejidad Social UC3M-UV-UZ (UMICCS), Madrid, Spain; 3 Institute for Biocomputation and Physics of Complex Systems (BIFI), University of Zaragoza, E-50018 Zaragoza, Spain; 4 Department of Economics, University College London, London, UK; 5 Information Systems Department, Faculty of Business and Economics, University of Lausanne, CH-1015 Lausanne, Switzerland; 6 UC3M-BS Institute for Financial Big Data (IFiBiD), Universidad Carlos III de Madrid, 28903 Getafe, Madrid Spain

## Abstract

Reputation plays a key role among the mechanisms supporting cooperation in our society. This is a well-known observation and, in fact, several studies have shown that reputation may substantially increase cooperation among subjects playing Prisoner’s Dilemma games in the laboratory. Unfortunately, recent experiments indicate that when reputation can be faked cooperation can still be maintained at the expense of honest subjects who are deceived by the dishonest ones. As experimental work is limited due to financial and other reasons, we present here an agent-based simulation model inspired by, and calibrated against, the results obtained in the experiment. We thus simulate much larger population sizes over longer times, and test other model parameters to see whether the observed behavior generalizes in those yet untried conditions. The results show that the collective behavior is qualitatively similar in larger systems and stable over longer times horizons. We conclude that the findings of the experimental work are meaningful, taking into account that the model is strictly tailored to our particular experimental setting and therefore it is a possible explanation of our observations whose applicability to other contexts requires further research. We argue that simulations like the ones presented here may also be useful to cheaply and quickly suggest settings and options to enhance and facilitate further experiments, which, in turn, may provide new tests of the models themselves.

## Introduction

Reputation is one of the most important mechanisms that allow cooperation to evolve and stabilize in social interactions. Building and maintaining a good reputation is key in this respect because it encourages trust and socially responsible behavior. For reputation to be useful, it has to take the form of some “marker” or some public information that characterizes a particular individual and must be easily recognized and interpreted by others. In contrast to direct *reciprocity*, which requires repeated interactions between the same people to support cooperation, reputation is an indirect mechanism that relies on an individual’s previous behavior with other individuals. This behavior is somehow made public knowledge in different forms and thus requires communication and information capabilities. Thus, when encountering another previously unseen person, individuals can act on the basis of the reputation of the former. Cooperation is costly but helps build a good reputation which, in turn, may lead to more cooperative acts towards oneself and to a better functioning society as a whole. While direct reciprocity is at work in small groups and organizations in which people meet frequently and repeatedly, reputation is a more general indirect mechanism that may work in larger and/or anonymous groups.

Cooperation based on reputational knowledge, or indirect reciprocity, has long been studied through theoretical models^1–6^, as well as experimentally^7–13^. In the present study we are particularly concerned with interacting populations that take the form of a social network in which each individual has a certain number of primary neighbors. In this context, it is crucially important that individuals in the network have control over which partners they interact with. In other words, they must be able to form new links and severe unwanted ones based on the availability of information about the actions of current and possible partners. If this is the case, cooperation may evolve and maintain itself to a remarkable degree thanks to positive assortment among cooperators. This has been convincingly shown in theoretical models and numerical simulations^14–20^ and, most importantly, by recent experiments with human subjects^11–13,21–24^. Of particular relevance for us here are the experiments reported in references^11–13^ which examine the interplay of the dynamical network factor and of the reputation knowledge on the amount of cooperation. These studies conclude that it is reputation that plays the most important role in the evolution of cooperation in the population.

The previous discussion and the cited research both assume that reputation is perfectly reliable and truly reflects the behavior of an individual, e.g., under the form of a list of individual’s actions extending in the past for a given length. However, in the real world this information can be manipulated in various ways, leading to uncertainty about the true reputation an individual is worth. Manipulating one’s reputation is difficult as long as individuals interact face-to-face in small groups where unfair behavior is simpler to spot and very detrimental to an individual if discovered. It has been recently shown that indirect reciprocity in groups fails to work if only an image scoring is available and there is uncertainty about individual reputation^25^. In the modern society many social and commercial interactions take place through communication networks^26^ and a variety of social media. In most instances, such interactions involve people who know each other only through an online identity^27^, without any connection whatsoever in the physical world. This makes manipulating a piece of information such as an individual’s reputation easier and much more likely, while, on the other hand, it affects many more people as the interactions in the digital world take place with larger numbers of subjects.

In a recent experiment we framed this question in a simplified environment as a dyadic Prisoner’s Dilemma (PD)^28,29^ in which participants were allowed to modify their reputation by paying a cost^13^ and, critically, they had no way to know whether another player’s reputation was true or fake.

Our experiment highlighted interesting behaviors and collective emergent phenomena although the results from the laboratory are still limited in several ways. Due to their high cost and the time and organization they take it is very difficult to go beyond the study of only a few experimental conditions at best, which means that the influence of the variation of several parameters cannot be studied in practice. It is also the case that the number of participants is usually severely limited to a few tens owing to the classroom sizes of the typical laboratory. There have been recent advances on this last point and it is now possible to run experiments with hundreds, or even thousands, of participants by using suitable web-based interfaces (see, e.g.^30^) but this is not yet widespread practice. Besides, experiments with large populations have problems of their own and the exploration of the parameter space remains out of the question.

In view of this situation we argue here that experiments can be usefully complemented and calibrated by numerical simulation models. However, by this we do not mean the standard theoretical models based on replicator dynamics ideas and on microscopic strategy revision rules like payoff-based or imitation-based^31^, which turn out to be mostly inapplicable to complex situations like the one studied in our experiment. Rather, we think of suitable numerical versions of the actual behavioral strategies that people use when playing in the laboratory. So, in our view, experiments and computer simulations go hand in hand, with experiments suggesting suitable behavioral models and simulations extending the domain of exploration of the parameter space that cannot be reached by experiments alone. In turn, numerical simulation results can also suggest new experiments or experimental settings that would have been difficult to design without that knowledge.

To make the article self-contained and for the sake of the reader, we first summarize the experimental setting in the next section. In the rest of the paper we present the numerical model that has been designed starting from the experimental results and its application to a more complete study of the model parameters. We conclude with the discussion of the obtained results and some suggestions for further work.

## Summary of the Experimental Setup

In our experimental sessions seven groups of twenty subjects connected in a social network played a Prisoner’s Dilemma game with their neighbors^13^. In this two-person game, players must decide whether to cooperate (C) or to defect (D). Similarly to several recent experimental settings (e.g.^11,21–23^), the chosen action is the same with all neighbors. Note that if actions could be chosen independently for each neighbor the network structure becomes almost irrelevant and the system turns to a collection of independent pairwise games. If both agents cooperate, each receives a payoff *R*. If one defects and the other cooperates, the defector receives *T* and the cooperator receives the payoff *S*. If both defect, each receives *P*. Since *T* > *R* > *P* ≥ *S*, defection is a dominant strategy and a rational payoff-maximizing player will choose to defect, although mutual cooperation yields a higher collective payoff, whence the dilemma. Subjects played a weak PD game (*P* = *S*) with their immediate neighbors with *T* = 10, *R* = 7, *P* = 0, and *S* = 0. Payoff values are the same as those used in^11^, where it was shown that the possibility to rewire links allows for cooperation to emerge when information about past actions of others, which amounts to their reputation, is available. The initial set of connections between the participants was chosen to be a regular lattice of degree 4. Participants played 30 rounds of the sequence described below. See^13^ for more details on the experimental protocol.

The reputation of a player was expressed through a *cooperation index α* which is the number of times the player has cooperated in the last five moves, thus *α* ∈ [0, 5]. We considered two treatments: a baseline one, called *Real Reputation* (RR) in which the cooperation index cannot be manipulated, and a modified one in which participants were informed that all of them were allowed to vary their cooperation index by paying a cost, called *Fake Reputation* (FR). At the beginning, all players receive an initial *α* of 3 based on the actions sequence *CDCDC*. Note that this form of reputation is related to but different from the one used in^11^ where explicit past choices of each player were available to all others. In contrast, in our experiment there is some uncertainty about the current behavior of a player even in the RR treatment. This uncertainty comes about because only the number of cooperative actions of the current first neighbors and potential partners is known, but not their order. In addition, neighbors are just unlabeled anonymous individuals who cannot be recognized from one round to the next. As a result, only an average success rate of interactions with other unspecified participants is provided.

In the Real Reputation (RR) treatment each round consisted of the following four stages: (i) action choice; (ii) neighborhood modification; (iii) link acceptance decision; (iv) feedback on payoffs. In the first stage, players receive information on the cooperation index of their current neighbors and have to select one of two actions. In the second stage, participants may decide to unilaterally suppress a link with a neighbor and they are also given the option to offer a link to a new, randomly chosen partner; in both cases, they only know the *α* value of the corresponding subject. In the following stage, participants see all link proposals from other players (and their *α*), which they can either accept or reject. After these decision stages a new network is formed, and subjects accumulate their payoff by playing the PD game in pairs with their current neighbors. They are neither informed about their neighbors’ payoffs nor about their neighbors’ individual current actions. Participants never know the full network topology.

The Fake Reputation (FR) treatment is identical to the RR treatment with the following fundamental difference: participants never know whether the observed cooperation index *α* of their partners is the real one or has been modified. Consequently, in this setup there is an additional stage between the first and the second stage of the RR treatment during which participants may choose to pay a cost in order to modify their *α* value. The chosen cost was 4 points per modified reputational point, per round. There is no cost if one just wants to show her true cooperation index.

## Model Description

### Initial setup

The initial configuration for the set of *N* agents is a random regular random graph of degree 4, which represents a dynamical network where edges can be created and removed during the model dynamics. The initial degree is chosen to be the same as in the experimental treatments in^13^. Every agent *i* has a cooperation index *α*_*i*_, that indicates how many times the agent cooperated in the last five rounds, that is, *α*_*i*_ ∈ [0, 5]. Cooperation indices are part of the information provided during the experiment at each round to each agent about their neighbors. The agents play a Prisoner’s Dilemma (PD) game with their neighbors using the same strategy against all of them, as described in the experimental setting. To compare with the results of the previous experiment, the payoff values have been chosen to be the same as in^13^, i.e., *T* = 10, *R* = 7 and *P* = *S* = 0.

### Agent dynamics

Following the experimental setting (see previous section and^13^), two model treatments have been considered: one in which the cooperation index cannot be manipulated (RR), and a modified one in which agents can change their cooperation index by paying a cost (FR). At the beginning, agents receive a random sequence of past actions of length five, so their initial cooperation index has an average value of 2.5 but it may be different for each of them. This is slightly different from the corresponding experimental setup but it is done to avoid the possibility of entering a loop of stereotyped behavior, given the greater regularity of the model evolution rules described below.

Mirroring the experiment, in the RR treatment, each round has four stages named: action choice, neighborhood modification, link acceptance, payoff feedback. These proceed as follows:

#### Action choice

Agents receive information on the cooperation index of their current neighbors, and select cooperation or defection as the action for all the PD games with their neighbors. Each agent computes the normalized average cooperation index of its neighbors as ${\hat{\alpha }}_{i}=\frac{1}{{k}_{i}}{\sum }_{j\in {\eta }_{i}}{\alpha }_{j}/5\in [0,1]$, where *k*_*i*_ is the number of neighbors of agent *i* and *η*_*i*_ is the set of agent *i*’s neighbors numbers. Then, the agent chooses to cooperate with probability *p*_*coop*_ = *F*${\hat{\alpha }}_{i}$, where *F* is a tunable parameter on the agent’s decision-making process.

#### Neighborhood modification

Agents may suppress, unilaterally, a link with the neighbor that has the worst cooperation index, and they can propose a link to a random agent, which was not already linked to them. The suppression of the link occurs with probability *p*_*cut*_, which is based on the complementary probability *p*_*accept*_ = 1 − *p*_*cut*_ of accepting a link. The probability of link acceptance, *p*_*accept*_, is based on the average cooperation index of the agent’s neighborhood, ${\hat{\alpha }}_{i}$, and on the cooperation index of the agent that has proposed the link, *α*_*j*_.

#### Link acceptance

Agents evaluate all the link proposals by seeing the corresponding agent’s cooperation index, *α*_*j*_, of all their potential neighbors. We assume that when *α*_*j*_ > ${\hat{\alpha }}_{i}$ we have *p*_*accept*_ = 1 and *p*_*accept*_ = 0 for *α*_*j*_ = 0. In all the other cases, when 0 < *α*_*j*_ < ${\hat{\alpha }}_{i}$ we have *p*_*accept*_ = *α*_*j*_/${\hat{\alpha }}_{i}$.

#### Feedback on payoffs

All agents receive their payments by accumulating payoffs from all the PD games in which they are involved.

The FR treatment is identical to the RR treatment but the agents never know if the observed cooperation index is the real one. Consequently, as in^13^, there is an additional stage between the first and second stage of the RR treatment. In that additional stage, agents can pay a cost in order to increase their *observable* cooperation index. This modification costs 4 points per increased point, as in^13^, and the purchased points are only valid for the round they are currently playing.

In the simulated FR treatment, we introduce a new kind of agent type, called *cheater*, to be defined below. The fraction of cheaters in the agent population is regulated by the parameter *f*_*ch*_ ∈ [0, 1], where *f*_*ch*_ = 1 stands for a population entirely composed by cheater agents. All the other agents are called *reliable*. A cheater agent defects with probability ${\rho }_{D}^{ch}\in [0,1]$ and it behaves as a reliable agent with probability $1-{\rho }_{D}^{ch}$, i.e. cooperates with probability *p*_*coop*_ = *F*${\hat{\alpha }}_{i}$. Whenever a cheater agent *i* has a cooperation index smaller than its neighbourhood average cooperation index, that is, *α*_*i*_ < ${\hat{\alpha }}_{i}$, it purchases reputational points for that round until *α*_*i*_ ≥ ${\hat{\alpha }}_{i}$. On the other hand, reliable agents purchase reputational points until *α*_*i*_ ≥ ${\hat{\alpha }}_{i}$ with probability ${\rho }_{R}^{rel}\in [0,1]$ and with probability $1-{\rho }_{R}^{rel}$ they keep their cooperation index unchanged.

For the sake of clarity, the main variables and parameters of the model are summarized, with their meanings, in Table 1.

**Table 1:** Variables and parameters of the model and their meanings.

*α*_*i*_	cooperation index of agent *i*
${\hat{\alpha }}_{i}$	average cooperation index of agent *i*’s neighbors
*F*	factor on agents’ decision (model parameter, [MP])
*p*_*coop*_ = *F*${\hat{\alpha }}_{i}$	probability of cooperation for reliable agents
*p*_*accept*_	probability of accepting a new link
*p*_*cut*_ = 1 − *p*_*accept*_	probability of cutting a link to a neighbor
*f*_*ch*_	fraction of cheater agents in the population (MP)
${\rho }_{D}^{ch}$	probability of direct defection for cheater agents (MP)
${\rho }_{R}^{rel}$	probability of purchasing reputation for reliable agents (MP)

## Results

**Figure 1:**
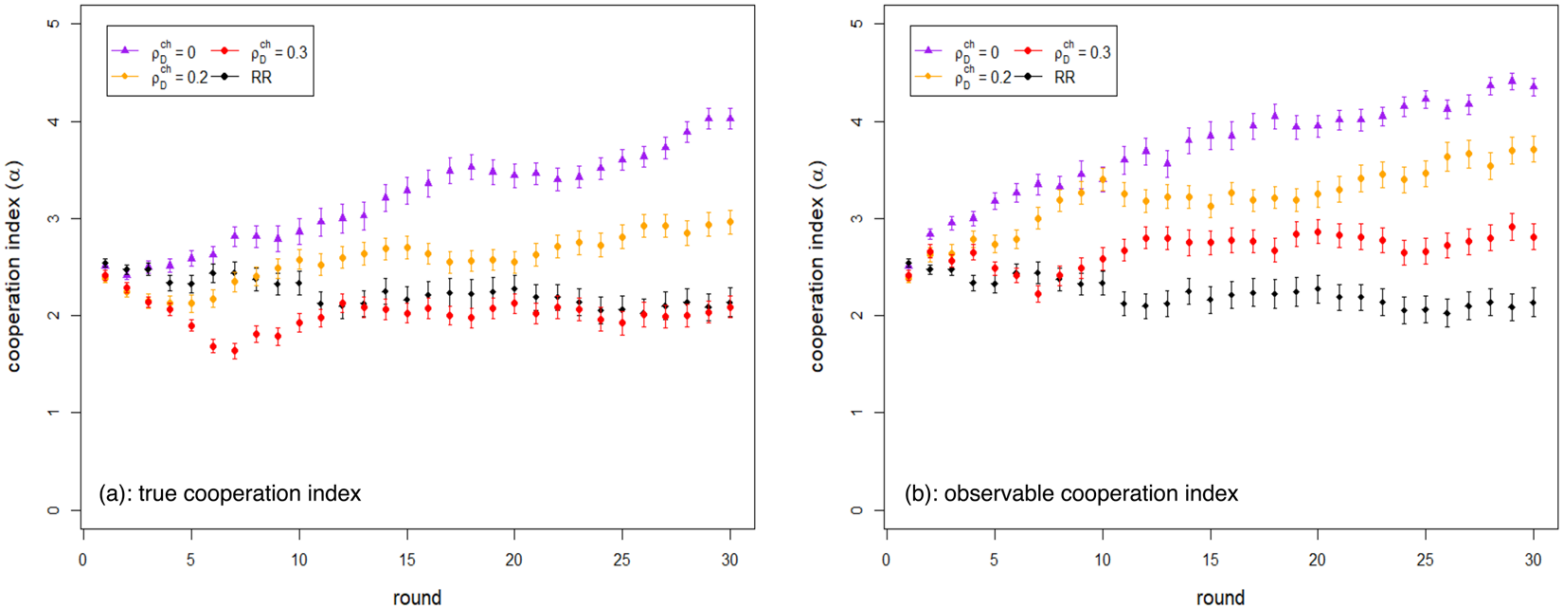
Simulated cooperation indices for cheaters’ behavior. Cooperation index as a function of the rounds of play in the simulated RR and FR model. (**a**) real cooperation index evolution; (**b**) observable cooperation index evolution. Results are averages of 25 runs. Standard deviations are shown as error bars.

We present the results of numerically simulated systems for the RR and FR treatments using the same number of agents (20) as in the laboratory experiments^13^, and adopting the agent update rules described in the previous section. The maximum number of rounds we simulated in this study was 100, instead of the 30 used in the experimental setting, to check the stability of our results with a longer time horizon. We have studied a wide range of values for all the model parameters: *f*_*ch*_, ${\rho }_{D}^{ch}$ and ${\rho }_{R}^{rel}$. We have also considered the effect of a damping term on the action decision making process varying the parameter *F*. For the FR treatment, the initial fraction of cheater agents in the population was chosen to be *f*_*ch*_ = 0.5, that is, half of the population plays as a cheater agent while the other half as a reliable one. The value *f*_*ch*_ = 0.5 is similar to what we empirically measured in^13^. Also, according to experimental results, we use ${\rho }_{R}^{rel}=0.25$, that is, the probability that a reliable agent purchases reputational points. We then investigate system dynamics for ${\rho }_{D}^{ch} < 0.5$ and *F* = 0.95 and 1. Figure 1 shows how the cooperation index evolves as a function of the round number during the simulations for different values of ${\rho }_{D}^{ch}$ and for *F* = 0.95. The RR model, i.e. ${f}_{ch}=0,{\rho }_{R}^{rel}=0,F=0.95$, is also shown for comparison. Results for *F* = 1 are similar and have been omitted. The observable cooperation index (Fig. 1, right image) reaches higher values, as it intuitively should, and similarly to what was observed in the experiment^13^.

The model parameter selection has been made after comparing the simulation results with the ones in^13^ and selecting those values that give the aggregate behavior that appears to be closer to the experimental results. We found that the most suitable choices were: ${\rho }_{D}^{ch}=0.3$, ${\rho }_{R}^{rel}=0.25$, *f*_*ch*_ = 0.5, and *F* = 0.95.

### Cooperation index

**Figure 2:**
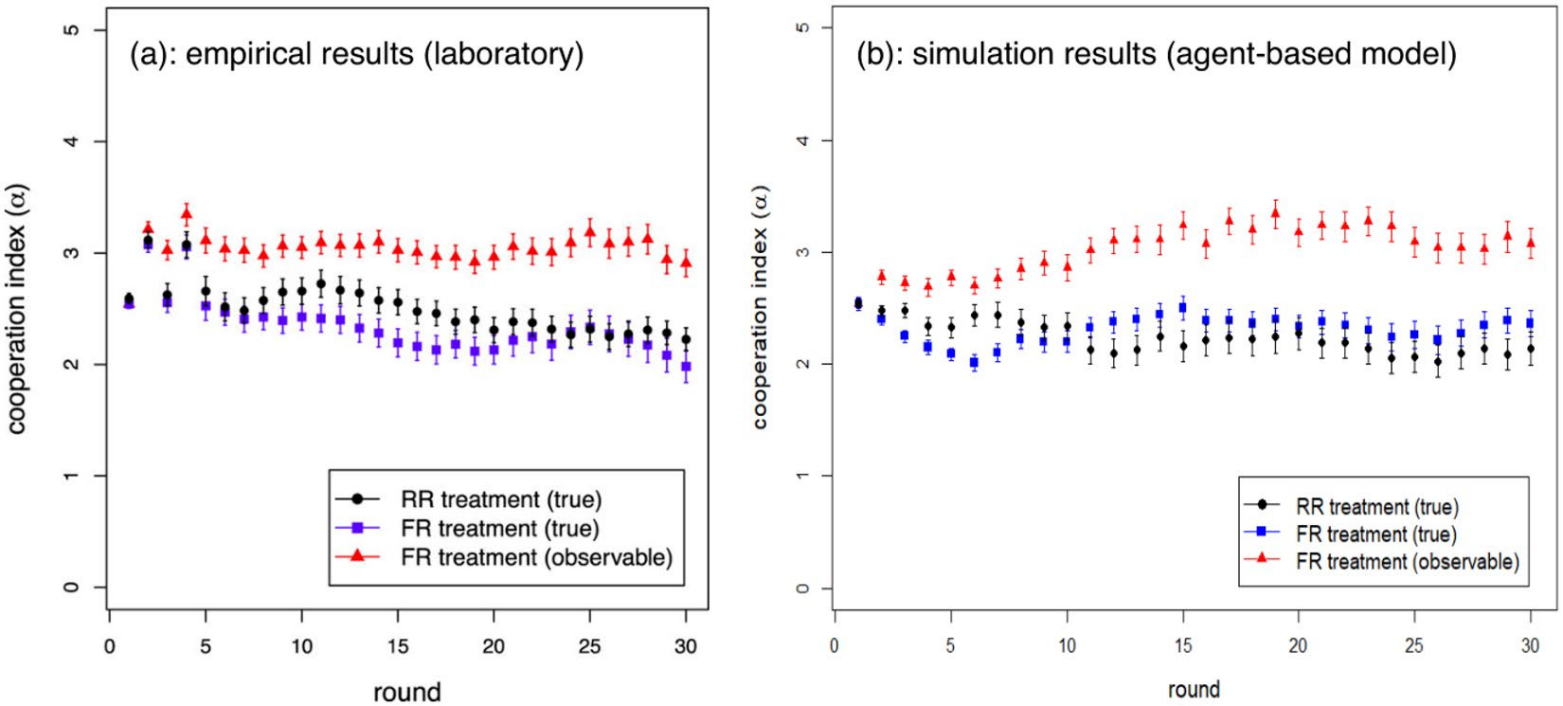
Comparison between empirical and simulated results. Cooperation index as a function of round. Left image: average experimental results^13^. Right image: numerical simulation results. Results are averages over 25 repetitions. The RR parameter values are: *f*_*ch*_ = 0, ${\rho }_{R}^{rel}=0$; while FR ones are: *f*_*ch*_ = 0.5, ${\rho }_{D}^{ch}=0.3$, ${\rho }_{R}^{rel}=0.25$.

Simulated results are compared with empirical ones in Fig. 2 adopting *F* = 0.95. The results are quite close to the experimental ones. It can be observed that, for the chosen parameter set, a difference of about half a point exists between real and visible cooperation in the FR treatment. Of course, the similarity between experimental and simulated results is not surprising: it was expected since we chose parameter values in the model that were suggested by the experimental results. Indeed, our goal is not to have generic agents that collectively behave as the real ones, which would be almost hopeless, but rather “statistical” agents that individually resemble the human ones that took part in the experiment in their decison-making behavior. In other words, the intention here is not to “explain” the observed behavior. On the contrary, we assume this behavior in order to enhance by simulation the limited range of the experimental settings.

### Purchased points

**Figure 3:**
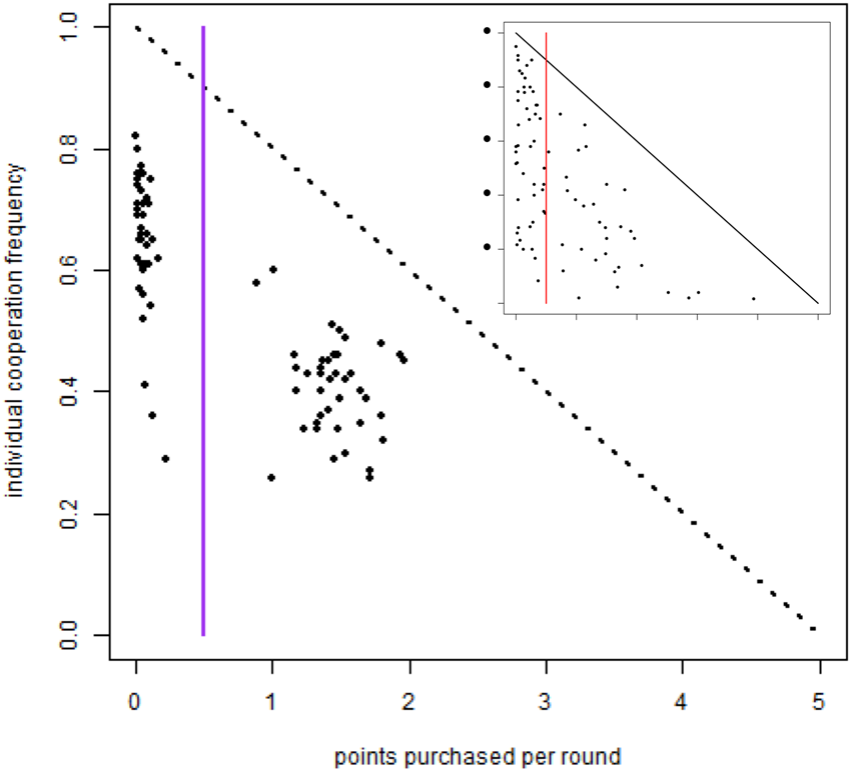
Comparison of individual players’ average behavior (cheating/cooperation). Each individual is represented by a dot whose abscissa gives the average number of points purchased per round and whose ordinate represents the individual’s cooperation frequency averaged over all rounds in the simulation run. The inset panel reports the same data from the experiment in^13^. Parameters values are the same as those of Fig. 2 (*f*_*ch*_ = 0.5, ${\rho }_{D}^{ch}=0.3$, ${\rho }_{R}^{rel}=0.25$, *F* = 0.95).

As said above, the FR treatment is characterized by the fact that players, unknown to the others, are allowed to purchase reputation points at each round. A useful view of the individual’s behavior is given by a plot in which each individual is represented by a dot. The x-coordinate of a given individual is the average number of points she has purchased per round during the run; the y-coordinate of the individual gives her cooperation frequency during the run. This is what is depicted in Fig. 3. The inset panel in this figure represents the same data for the experimental results. The vertical line is an arbitrary (but, as can be seen from the plot, reasonable, in so far as there are seemingly two different groups of subjects) threshold that separates players that buy less than half a point per round in average, from those that buy more than half a point. For the sake of clarity, we recall here that we dubbed the first group of players “reliable”, while the others were called “cheaters”. This binary classification is a simplification but it allows us to group behaviors instead of treating them as a continuous variable. This is useful to understand the system behavior in terms of well-defined behavioral types and gave useful results when applied to the experimental data^13^.

Given that we introduced an amount of cheater agents approximately equal to the experimentally observed quantity (*f*_*ch*_ = 0.5), it is again not surprising that the simulated population behavior is qualitatively similar to the experimental results with human subjects. It can be observed, however, that the simulation results are more concentrated, a phenomenon that can be attributed to the average artificial agent behavior compared to the more idiosyncratic human players which have a more spread-out distribution in the scatter plot. In both cases, cheaters cooperate less on average.

### Frequency of cooperation

**Figure 4:**
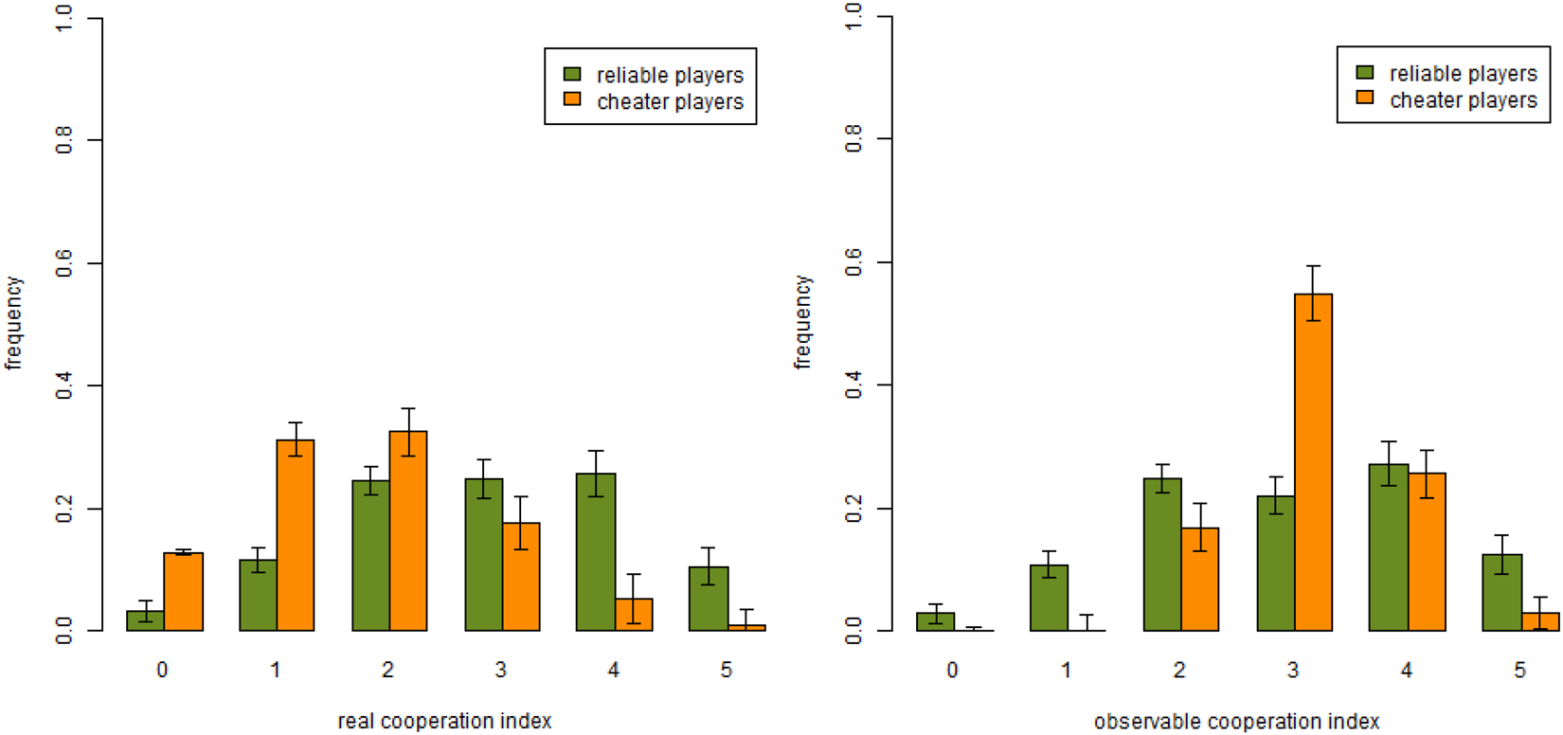
Simulated cooperation indices per individual type in small systems. Frequency of reliable and cheater players for real (left) and observable (right) cooperation index in the simulated FR treatment. Results are averages over 25 runs. Parameters values are the same as those of Fig. 2 (*f*_*ch*_ = 0.5, ${\rho }_{D}^{ch}=0.3$, ${\rho }_{R}^{rel}=0.25$, *F* = 0.95).

We continue the comparison between human agents and artificial agents behavior by showing the histograms giving the fraction of the population that has a given average cooperation index in the FR treatment for reliable and cheater players respectively. This is shown in Fig. 4 where simulation results are reported. Comparing them with the laboratory results in^13^ one can see that the general patterns are similar in both cases, although the distributions for the simulated population are more centered between 1 and 2 for the true cooperation index distribution (left panel) and between 2 and 4 for observable cooperation index distribution. This is essentially a consequence of the less erratic behavior of the agents.

### Scaling up the population size

All the results shown until now were for a population size of 20, the same size that was used in the laboratory experiment^13^. This is interesting enough but one is let with the question of whether a larger number of participants would give rise to fundamentally different behavior. We want also to stress that human subjects were aware of the fact that they were playing against other people in the room, putting them in a situation of a small-scale experiment. However, the same experimental protocol can be easily extended to a larger population. We thus assume that participants’ incentives and consequent behavior should not completely change when playing in a larger pool of people. Managing a large number of subjects is difficult to do in a laboratory setting, although today there exist web-based systems that allow hundreds of people to participate in an experiment. Yet, those experiments are hard to set up, control, and analyze, not to speak of the financial aspects involved. Thus, numerical simulation provides a cheap means to explore untried possibilities.

**Figure 5:**
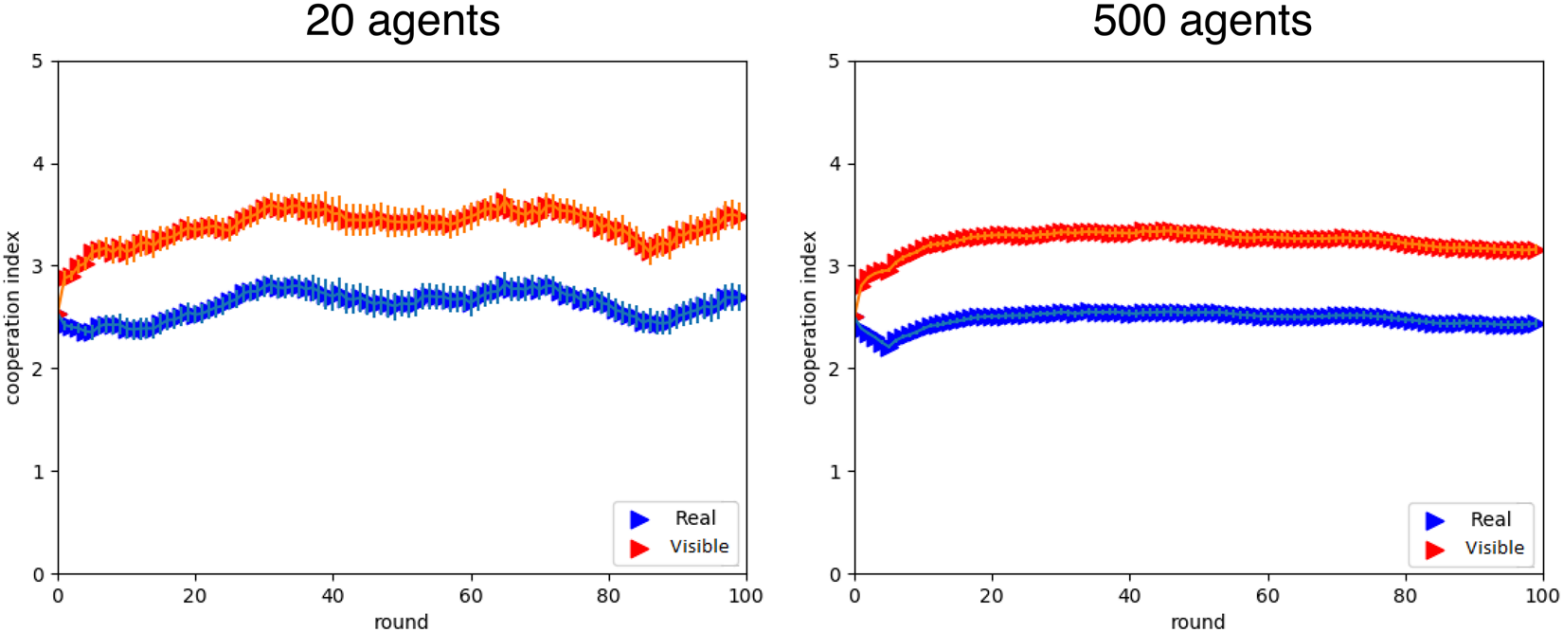
Size and time horizon effects. Comparing average cooperation in populations of size 20 (left) and size 500 (right). Blue curves: real cooperation index. Red curves: visible cooperation index. Parameters values are the same as those of Fig. 2 (*f*_*ch*_ = 0.5, ${\rho }_{D}^{ch}=0.3$, ${\rho }_{R}^{rel}=0.25$, *F* = 0.95). Results are averages over 25 runs of the simulated system.

In what follows, we report results for simulations performed with 500 agents that interact during 100 rounds in the simulated FR treatments. Figure 5 depicts the average cooperation results. Compared with the laboratory results for 20 participants (left panel), it is clear that the trend is maintained and the fluctuations are lower in the larger simulated population. In particular, it is reassuring to see that nothing odd happens when more players interact during more rounds; rather, the behavior becomes more stable and statistically reliable (right panel). We also conducted simulations with 1000 agents with basically the same results that we omit for the sake of brevity.

**Figure 6:**
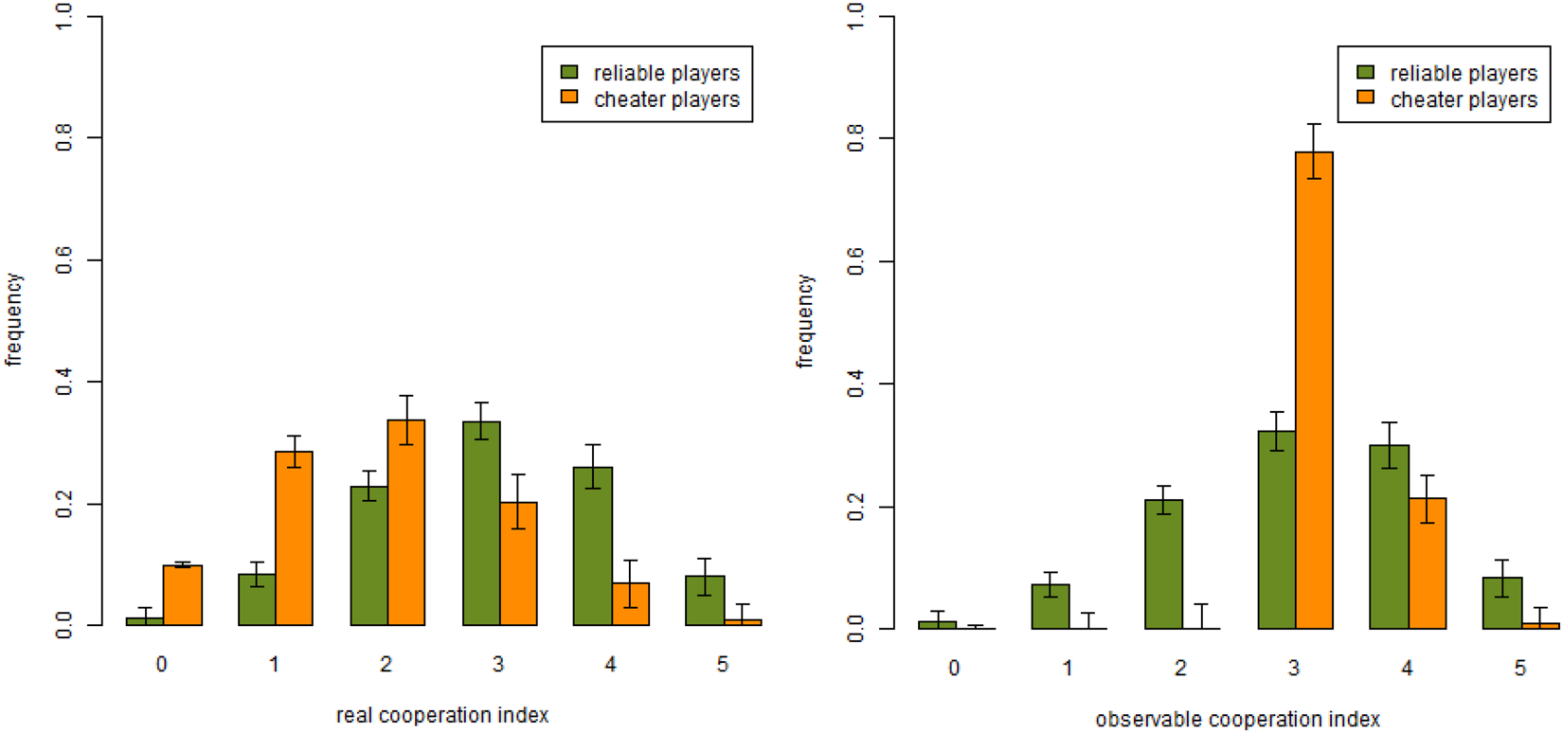
Simulated cooperation indices per individual type in larger systems. Real (**a**) and visible (**b**) cooperation index frequency for reliable and cheater players in the large population, i.e. 500 agents. See also Fig. 4 for comparison with the same results on the small simulated system. Parameters values are the same as those of Fig. 2 (*f*_*ch*_ = 0.5, ${\rho }_{D}^{ch}=0.3$, ${\rho }_{R}^{rel}=0.25$, *F* = 0.95). Simulation data are averages over 25 runs.

Now, comparing player type frequencies in the large populations in the FR treatment, we find very similar trends for the real cooperation index, as shown in Fig. 6 (left panel), while visible cooperation frequencies (right panel) seem to experience a shift towards the right of the x-axis and cheaters essentially stay around cooperation index 3, instead of being mainly distributed between indices 3 and 4 as in the smaller population (see Fig. 4).

**Figure 7:**
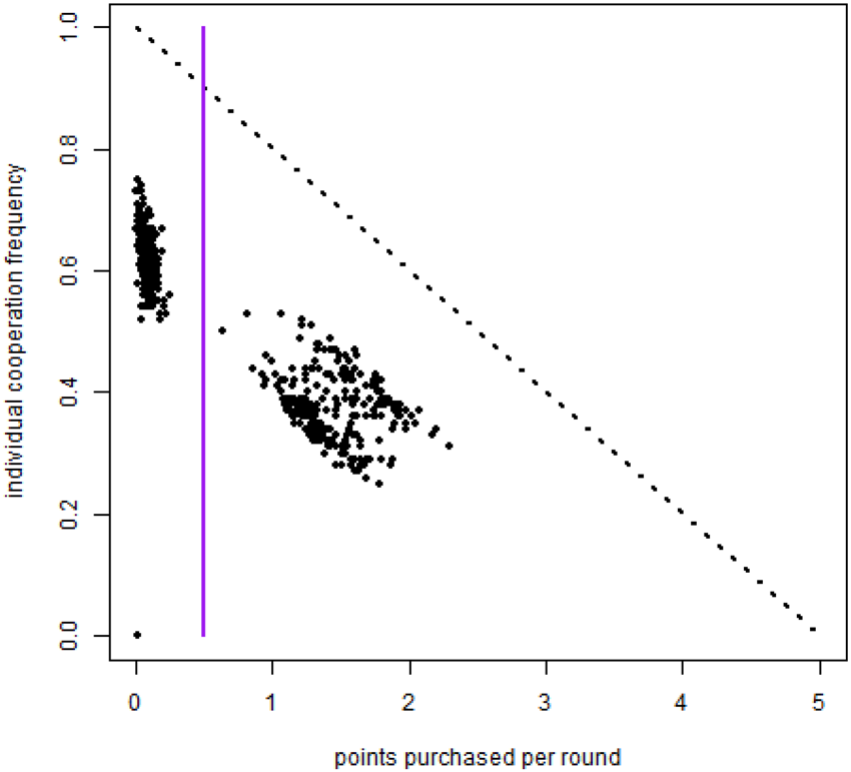
Individual players’ average behavior (cheating/cooperation) in larger systems. As in Fig. 3, we represent individuals by the number of points they purchased per round (x axis) and their cooperation frequency (y axis), for a population of 500 agents in a simulated FR treatment. Parameters values are the same as those of Fig. 2 (*f*_*ch*_ = 0.5, ${\rho }_{D}^{ch}=0.3$, ${\rho }_{R}^{rel}=0.25$, *F* = 0.95).

In the following Fig. 7 we show the scatterplots of the points purchased per round by each individual against the individual’s cooperation frequency in large simulated populations. The correlation patterns are similar to what happens for smaller system sizes (see Fig. 3): we observe cheaters cooperating less in the average but that in the large population case the density of points in the two regions is much higher and points are less scattered around. Thus, it appears that using more agents in the simulations really gives crisper and more stable patterns of behavior.

Regarding the evolution of the degree distribution we have noticed that in the small population case the network quickly saturates and becomes an almost complete graph. This behavior is very close to the trend observed in the experiment^13^. In the large population case the trend is the same and the degree growth rate is even faster in the large population.

From the previous results for larger populations we can infer that using more agents does indeed improve the stability of the dynamical systems and the associated statistics since there are far less fluctuations. Nevertheless, it is also clear that the small and the large systems basically show the same trends in all measured quantities, at least in the particular case studied here. This means that using the typical 15 to 25 participants in a laboratory experiment, numbers that are often dictated by logistic and financial limitations, does not seem to impair the qualitative nature of the results. On the other hand, if one can afford many more participants, the simulations suggest that the results are more stable and their statistical significance is higher.

## Conclusions

Our main objective in the present study was to design a numerical simulation model of a system where reputation can be faked, based on artificial agents since such a model can be suitably parameterized and can be run quickly and repeatedly, substituting the need for actual experiments. Our agent model does not try to faithfully reproduce the idiosyncrasies of particular human agents, it rather strives to represent the rules of typical agents such that the collective behavior of the agents’ interactions results in a global dynamics that is qualitatively in agreement with the experimental observations. To validate our model design, we first compared the numerical simulation results with the same system size as in the laboratory experiment. Having thus obtained a good qualitative fit, we then studied much larger systems over longer time horizons, that would make them either unfeasible or difficult to study in a laboratory with human participants. The main results we obtained is that larger populations essentially behave in the same qualitative manner as the small one, except that all results have smaller fluctuations, both because the populations are larger and also because one can easily and quickly perform many repetitions of the virtual experiment before taking the averages.

This result has two interesting implications. First, it justifies the use of classroom-size laboratory experiments, at least in this case, and suggests that people might behave in a large system by just keeping the links that have a reasonable observable reputation and making new links to similar ones, leading to a growth of the mean degree. This is a clear-cut prediction that raises the question as to whether real people, with limited attention and cognitive capabilities can actually behave in that manner. We envisage that the way information about the (very many) other participants can be key to the verification of this prediction. Second, large scale simulations suggest that the observation could exhibit less fluctuations compared to those of small size experiment. This seems to indicate that it could be better, in terms of the statistical significance of the results, to run a large system than many instances of smaller ones, something that again requires experimental verification. In this sense, it has to be kept in mind that, following the findings about static PD experiments^32^, we have proposed a model where payoffs do not play any role. It then goes without saying that it would be important to check the accuracy of this assumption by repeating the experiments in small size systems with different payoffs; if the model is valid in these other setups, we would then have a very general manner to describe quite a large range of experiments.

Another point about the comparison of our model with other experimental setups relates to one of the findings of our large scale simulations, namely the rapid growth of the number of links in the system. Such large growth rates are possible only because link creation and deletion are free in the model. However, actual socio-economic networks have mean degrees that do not exceed 15 in most measured cases (see, for instance^33^). This is because in the real social world link creation, and even link cutting, are not free. They often imply a cost, either economical or of other types. Moreover, issues such as time and attention span limitations prevent actors to engage in too many simultaneous contacts. It would certainly be interesting to modify the network dynamics part of our model so as to take these factors into account. Likewise, concepts such as the degree distribution function, the mean distance, or the clustering coefficient would not make much sense for our very dense final population graphs. However, our main purpose here was to create a numerical counterpart of the experimental setting we used in^13^. The experiments we are proposing here would allow us to extend our model to those, more realistic situations.

To conclude, we stress that, by design, the main limitation of our approach is that it cannot be applied to other situations as it has been purposedly tailored to the setting described in the experiment. On the other hand, using general game-theoretical models such as learning or replicator dynamics would certainly have prevented us from approximately matching the human behavior in the experiment. Another advantage of the specialized agent system is that simulations may also be used to suggest further experiments to be tried out as we have done above. We are thus led to argue that the parallel use of experiments with people and of suitably designed agent simulations greatly enhances the scope of both laboratory experiments and agent-based simulations. Eventually, the interaction of models and experiments should lead to a better understanding of the behavior of a large class of people, and the discrepancies could be classified by looking at the differences with this average behavior. That would be a real contribution to advancing the behavioral sciences. We hope that the success of the model we are presenting here stimulates further work along these lines.

## References

[1] Nowak MA, Sigmund K (1998). Evolution of indirect reciprocity by image scoring. Nature.

[2] Panchanathan K, Boyd R (2004). Indirect reciprocity can stabilize cooperation without the second-order free rider problem. Nature.

[3] Brandt H, Sigmund K (2005). Indirect reciprocity, image scoring, and moral hazard. Proceedings of the National Academy of Sciences.

[4] Nowak MA, Sigmund K (2005). Evolution of indirect reciprocity. Nature.

[5] Ohtsuki H, Iwasa Y (2006). The leading eight: social norms that can maintain cooperation by indirect reciprocity. Journal of Theoretical Biology.

[6] dos Santos M, Rankin DJ, Wedekind C (2011). The evolution of punishment through reputation. Proceedings of the Royal Society of London B.

[7] Milinski M, Semmann D, Krambeck H-J (2002). Reputation helps solve the “tragedy of the commons”. Nature.

[8] Wedekind C, Braithwaite VA (2002). The long-term benefits of human generosity in indirect reciprocity. Current Biology.

[9] Engelmann D, Fischbacher U (2009). Indirect reciprocity and strategic reputation building in an experimental helping game. Games and Economic Behavior.

[10] Pfeiffer T, Tran L, Krumme C, Rand DG (2012). The value of reputation. Journal of the Royal Society Interface.

[11] Cuesta JA, Gracia-Lázaro C, Ferrer A, Moreno Y, Sánchez A (2014). Reputation drives cooperative behaviour and network formation in human groups. Scientific Reports.

[12] Gallo E, Yan C (2015). The effects of reputational and social knowledge on cooperation. Proceedings of the National Academy of Sciences.

[13] Antonioni A, Sánchez A, Tomassini M (2016). Cooperation survives and cheating pays in a dynamic network structure with unreliable reputation. Scientific Reports.

[14] Eguíluz VM, Zimmermann MG, Cela-Conde CJ (2005). & San Miguel, M. Cooperation and the emergence of role differentiation in the dynamics of social networks. American Journal of Sociology.

[15] Santos FC, Pacheco JM, Lenaerts T (2006). Cooperation prevails when individuals adjust their social ties. PLoS Computational Biology.

[16] Perc M (2006). Double resonance in cooperation induced by noise and network variation for an evolutionary prisoner’s dilemma. New Journal of Physics.

[17] Szolnoki A, Perc M, Danku Z (2008). Making new connections towards cooperation in the prisoner’s dilemma game. EPL (Europhysics Letters).

[18] Szolnoki A, Perc M (2009). Resolving social dilemmas on evolving random networks. EPL (Europhysics Letters).

[19] Perc M, Szolnoki A (2010). Coevolutionary games - A mini review. Biosystems.

[20] Perc M, Gómez-Gardeñes J, Szolnoki A, Floría LM, Moreno Y (2013). Evolutionary dynamics of group interactions on structured populations: A review. Journal of the Royal Society Interface.

[21] Rand DG, Arbesman S, Christakis NA (2011). Dynamic social networks promote cooperation in experiments with humans. Proceedings of the National Academy of Sciences.

[22] Wang J, Suri S, Watts DJ (2012). Cooperation and assortativity with dynamic partner updating. Proceedings of the National Academy of Sciences.

[23] Fehl K, van der Post DJ, Semmann DJ (2011). Co-evolution of behavior and social network structure promotes human cooperation. Ecology Letters.

[24] Antonioni A, Cacault MP, Lalive R, Tomassini M (2014). Know thy neighbor: Costly information can hurt cooperation in dynamic networks. PLoS ONE.

[25] Nax H, Perc M, Szolnoki A, Helbing D (2015). Stability of cooperation under image scoring in group interactions. Scientific Reports.

[26] Rainie, L. & Wellman, B. *Networked. The New Social Operating System* (*MIT Press, Cambridge, MA*, 2012).

[27] Kendall, L. *The Handbook of Internet Studies* (*Wiley-Blackwell*, 2011).

[28] Rapoport, A. & Chammah, A. M. *Prisoner’s Dilemma* (*University of Michigan Press, Ann Arbor*, 1965).

[29] Axelrod, R. *The Evolution of Cooperation* (*Basic Books*, 1984).

[30] Rand DG (2012). The promise of mechanical turk: How online labor markets can help theorists run behavioral experiments. Journal of Theoretical Biology.

[31] Weibull, J. W. *Evolutionary Game Theory* (*MIT Press, Boston, MA*, 1995).

[32] Grujić J (2014). A comparative analysis of spatial prisoner’s dilemma experiments: Conditional cooperation and payoff irrelevance. Scientific Reports.

[33] Newman, M. E. J. *Networks: An Introduction* (*Oxford University Press*, *Oxford, UK*, 2010).

